# An Atypical Case of Marchiafava-Bignami Disease in a Young Chronic Alcoholic: Challenges in Diagnosis and Prognosis

**DOI:** 10.7759/cureus.75468

**Published:** 2024-12-10

**Authors:** Devika Menrai, Shyam Kiran Gandam Venkata, Sudeep Chakravarthy Bhuram, Sai Sruthi Bhuram

**Affiliations:** 1 Critical Care Medicine, Springfield Clinic, Springfield, USA; 2 Pulmonary and Critical Care Medicine, Southern Illinois University School of Medicine, Springfield, USA

**Keywords:** chronic alcoholism, confusion, malnutrition, marchiafava-bignami disease, wernicke encephalopathy

## Abstract

A 27-year-old male patient with chronic alcohol use disorder was diagnosed with Marchiafava-Bignami disease (MBD) after experiencing an episode of unconsciousness. MRI scans revealed lesions in the corpus callosum and adjacent white matter. Despite prompt initiation of intensive treatment with high-dose thiamine and corticosteroids, the patient only partially recovered, remaining disoriented and exhibiting persistent neurological deficits during follow-up. MBD is a rare condition seen in individuals with chronic alcohol use disorder and malnutrition, often resulting in unfavorable outcomes despite prompt intervention. This case underscores the impact of the patient’s young age at the onset of alcohol dependence, a low Glasgow Coma Scale score, and the presence of extracallosal lesions on his overall prognosis. We discuss the pathophysiology, presentation, and treatment. We also describe the role of each prognostic factor of MBD in an unfavourable outcome highlighting the need for a larger study to define various prognostic indicators at presentation and identify appropriate treatment approaches.

## Introduction

Marchiafava-Bignami disease (MBD) is a rare neurological disorder characterized by demyelination and necrosis of the corpus callosum, resulting from chronic alcoholism and malnutrition. First described in 1903 by Italian pathologists Amico Bignami and Ettore Marchiafava, the disease was originally linked to the consumption of inexpensive Chianti wine. However, it is now understood to be associated with any form of chronic alcohol abuse, often exacerbated by malnutrition and thiamine deficiency. To date, only about 300 cases have been reported worldwide, making it a severely underrecognized condition. Ethanol-induced neurotoxicity, oxidative stress, and severe vitamin deficiencies affect myelin-rich areas such as the corpus callosum and other white matter regions [1,2]. The clinical presentation of MBD can vary widely, from acute cases with rapid deterioration to more insidious, chronic forms. Patients may present with seizures, cognitive impairment, motor dysfunction, altered levels of consciousness, mutism, and other neurological deficits. Early diagnosis, typically through magnetic resonance imaging (MRI), is critical to prevent irreversible damage [3,4]. However, the prognosis remains guarded, with several patients showing limited recovery despite prompt treatment [5].

We present a case report of MBD diagnosed following a binge-drinking episode. The patient’s young age, critical neurological condition, and the presence of extracallosal lesions contribute to a unique and challenging clinical scenario. It offers insight into the complexities of this rare condition and its prognostic factors.

## Case presentation

A 27-year-old male patient with a 15-year history of chronic alcohol use disorder, with daily consumption of varying quantities of whiskey and no other comorbidities, was brought to the emergency department following a binge-drinking episode for sweating and loss of consciousness for an unknown duration. On examination, he was afebrile, had stable hemodynamics, appeared cachectic with a Body Mass Index (BMI) of 16.5 kg/m2, drowsy, disoriented, and exhibited scleral icterus. A neurological assessment revealed a Glasgow Coma Scale (GCS) score of 6, spasticity in both upper and lower limbs, and a positive Doll’s eye reflex.

Laboratory tests indicated transaminitis with aspartate aminotransferase (AST) of 111.8 U/L (normal range <35 U/L), alanine aminotransferase (ALT) of 57.5 U/L (normal range <45 U/L), alkaline phosphatase (ALP) of 61 U/L (normal range 53-128 U/L) and total bilirubin of 1.2 mg/dl (normal range 0.2-1.2 mg/dL). Magnetic resonance imaging (MRI) of the brain showed bilaterally symmetrical hyperintensities on T2-weighted images, hypointensities on T1-weighted images, and diffusion restriction in the splenium of the corpus callosum and periventricular white matter in the parieto-occipital lobe, findings highly suggestive of MBD. The patient was admitted to the intensive care unit (ICU) and treated with high-dose intravenous thiamine 500 mg every eight hours, intravenous dexamethasone 4 mg every 12 hours, and multivitamin supplements. He required nasogastric tube feeding due to the risk of aspiration, but he did not require intubation. Over the course of hospitalization including 10 days in the ICU and two weeks on the general medicine floors, his GCS improved to 10. The patient was discharged with home physical therapy.

At the four-month follow-up, despite GCS improvement, he remained disoriented to time, place, and person. His family reported sleep disturbances and episodic anger outbursts. Neurological examination revealed bilateral upper limb weakness, more pronounced on the left, with resting tremors. He also required a chronic urinary catheter due to urinary retention caused by a neurogenic bladder. Despite alcohol cessation and nutritional supplementation, the patient showed minimal improvement in symptoms at the one-year follow-up.

## Discussion

MBD is a rare neurological disorder predominantly seen in chronic alcoholics, characterized by demyelination and necrosis of the corpus callosum, most commonly observed in males between the ages of 40 and 60 years.

The pathophysiology of MBD is incompletely understood. It is believed to revolve around the effect of alcohol, which is metabolized by aldehyde dehydrogenase in the central nervous system, forming reactive oxygen species that result in oxidative stress [6]. Additionally, alcohol consumption causes thiamine deficiency, which leads to adenosine triphosphate (ATP) depletion, increased catecholamine neurotransmitters, and decreased synthesis of glutathione and myelin, all of which result in an inability to counter oxidative stress [1]. The corpus callosum, rich in myelin, is especially susceptible to degradation, correlating with total lifetime alcohol consumption [7,8]. This process results in cytotoxic edema in neurons, followed by a breakdown of the blood-brain barrier [9], necrosis, focal demyelination, and the formation of cystic lesions at a macroscopic level. Microscopic features include white matter necrosis, foamy histiocytes with CD68 and CD163 markers, gliosis, demyelination, necrosis, and other inflammatory cells [1].

Clinical features

MBD may present with varying combinations of neurological and psychiatric symptoms. Based on clinico-radiological evidence, the disease may be classified as MBD type A or type B, as differentiated in Table 1. Some patients may mimic the presentation of motor neuron disease [10], while some survivors may report long-term interhemispheric disconnection syndrome. This syndrome is characterized by a variety of motor and cognitive deficits, including split-brain phenomenon, impaired decision-making, hemialexia, unilateral apraxia, absence of interhemispheric transfer of stimulus from limbs unilaterally, and visual anomia [11,12].

**Table 1:** Clinico-radiological classification of Marchiafava-Bignami disease.

	MBD type A	MBD type B
Clinical presentation	Greater impairment of consciousness (stupor, coma)	Lesser impairment of consciousness
	Seizure	Behavioral disorders
	Pyramidal tract symptoms: hyperreflexia, spasticity, weakness	Gait disturbance
Radiological findings	Diffuse complete callosal involvement on MRI	Partial callosal involvement on MRI
Prognosis	Poor prognosis	Favorable prognosis

With such a wide array of presentations, it is often difficult to clinically suspect MBD, reinforcing the need for imaging studies to rule out other differentials, including Wernicke’s encephalopathy, sagittal sinus thrombosis, multiple sclerosis, hypoglycemic brain damage, and motor neuron diseases, characteristic signs of which are described in Table 2.

**Table 2:** Characteristic radiological findings of differential diagnoses of Marchiafava-Bignami disease. MRI: magnetic resonance imaging; FLAIR: fluid-attenuated inversion recovery; DWI: diffusion-weighted imaging.

Disease	Radiological finding
Wernicke encephalopathy	T2-weighted MRI shows high-intensity bilaterally symmetrical lesions around the third ventricle in the dorsomedial thalami and periaqueductal mid-brain.
Sagittal sinus thrombus	Unenhanced CT head shows: Cord sign: Hyperattenuated cord-like transverse sinus on axial view, Delta sign: Triangular dense vein typically seen in posterior superior sagittal sinus. Contrast-enhanced CT head shows: Empty delta sign: Triangular filling defect within the superior sagittal sinus with surrounding enhancement.
Multiple sclerosis	MRI with FLAIR shows lesions disseminated in space and time. Space: >= 1 lesion in >=2 characteristic locations: periventricular, juxtacortical, posterior fossa, spinal cord. Time: Simultaneous presence of gadolinium-enhanced and non-enhanced lesions at any time OR new T2-weighted gadolinium-enhanced lesion on follow-up MRI irrespective of the time of baseline imaging.
Hypoglycemic brain damage	DWI MRI shows hyper-intensities in the grey matter of the cortex, hippocampus, internal capsule, and basal ganglia with thalamic preservation.

Management

Early diagnosis with cranial MRI and initiation of treatment with parenteral thiamine and multivitamin is presumed to yield better outcomes for patients with MBD. The parenteral formulation can be transitioned to per oral treatment once GCS improves and the patient can tolerate it. Typically, MBD presents with a "sandwich sign" on MRI, described as a symmetrical T2-weighted hyperintensity lesion of the central corpus callosum sparing the dorsal and ventral layers [13]. There is a proven benefit of both physical and occupational therapy in survivors, aiming to restore motor capabilities, daily functionality, and their role in society. A study in Brazil indicated that physical and occupational therapy successfully resulted in a significant increase in motor functionality, but no change in cognitive ability in a survivor of MBD [14]. The study outlined an effective rehabilitation program where physical therapy focused on strength building, and stabilization of large joints, gross motor coordination, and gait training. Occupation therapy focuses on training the patient to carry out finer functions like writing, and developing independence in activities of daily living and using gait assist devices. An initial monthly follow-up with a gradual decrease in frequency is recommended to assess improvement [14].

Prognostic factors

Despite early intervention, patients with poor prognostic factors on presentation may succumb or make an incomplete recovery with lifelong neurological deficits [5]. There is, therefore, a critical need to study and understand the various factors responsible for poor outcomes, some of which we have discussed below and outlined in Table 3.

**Table 3:** Factors of poor prognosis in Marchiafava-Bignami disease (MBD). MAST score: Michigan Alcohol Screening Test score (a questionnaire usually filled by the physician used to determine the degree of alcohol dependence in an individual).

Factor	Criteria
Alcohol dependence	MAST score >= 6
Age	Early initiation of alcohol consumption
Clinical presentation	Glasgow Coma Scale < 10 on hospital admission
	Type A MBD: seizures, dysarthria, great deficit of consciousness
Radiology	Presence of extracallosal lesions on MRI Brain
	Complete corpus callosum degeneration on MRI Brain

Degree of Alcohol Dependence

The degree of alcohol dependence, as evaluated using the Michigan Alcohol Screening Test (MAST), has been shown to influence prognosis and disease outcomes. A MAST score ≥ 6 is associated with a severe drinking problem and a poor outcome in MBD. Our patient had a MAST score of 12, likely contributing to his poor recovery [15]. Surprisingly, the duration of alcoholism does not influence recovery or the lack thereof [16]. The total lifetime alcohol consumption over any duration determines clinical outcome.

Clinical Exam on Presentation

MBD can be classified into two clinical-radiological types: MBD-A (impaired consciousness, seizures, dysarthria, pyramidal tract syndromes, and diffuse corpus callosal swelling on MRI/CT) and MBD-B (cognitive disorder, gait disturbance, dysarthria, brain hemispheric disconnection, and partial corpus callosal lesions on MRI/CT), with the former being associated with a longer disease course and poorer outcome [17]. 

The GCS score at presentation plays a major role in predicting patient outcomes. A GCS score below 10 on admission indicates a poor clinical course. In this case, the patient's extremely low GCS score of 6 [16] on presentation indicates his poor prognosis.

Radiological Findings

MBD is a clinical-radiological diagnosis, with cerebral MRI findings playing a pivotal role not only in diagnosis but prognosis as well. MBD is characterized by demyelination and necrosis of part or all of the corpus callosum. A typical case of MBD will present with hyperintense T2-weighted/fluid-attenuated inversion recovery (FLAIR) and diffusion-weighted imaging (DWI) signals in the middle layer of the corpus callosum (the "sandwich sign") in the early stages of the disease [1]. In atypical MBD, extracallosal lesions may be found in any region of the brain's white matter, including periventricular white matter, as seen in this patient. Studies provide evidence that extracallosal lesions indicate lethal outcomes or permanent neurological deficits in survivors [3].

Age

In contrast to the average age of onset (40-60 years), the patient in our case was only 27 years old, an atypical age for MBD to present. As previously described, alcohol damages myelin-rich brain tissue and impairs neuronal plasticity. Early adolescence is a period of brain maturation marked by increasing white matter connections between different regions, allowing greater integration. By initiating alcohol consumption at the vulnerable age of 12, as was the case with our patient, not only is alcohol-seeking and dependence behavior enhanced, but developing connections are also severed, disrupting neuronal plasticity [18]. This leads to a rapid decline in gray matter and reduced growth of white matter volume compared to those who do not engage in heavy drinking [19], leaving lasting cognitive and behavioral deficits.

Outcome evaluation

Evaluating functional and neurological outcomes in alcoholism is essential for understanding the extent of cognitive and physical impairment. Two commonly used tools for this purpose are the Modified Rankin Scale (mRS), the Modified Oxford Handicap Scale (MOHS), and the Abbreviated Mental Test (AMT).

The mRS assesses functional independence, primarily used in stroke but also applicable in chronic conditions like alcoholism. Alcoholism, particularly in its advanced stages, can lead to significant neurological damage, including cerebellar atrophy and peripheral neuropathy, impairing motor coordination and independence. The mRS ranges from 0 (no symptoms) to 6 (death), helping clinicians gauge the severity of alcohol-related disability, often due to alcohol-induced strokes or liver-related encephalopathy. The MOHS, like the mRS, evaluates disability but offers a broader assessment of social and psychological functions. This is important for alcoholics, whose social and mental functioning often deteriorates with prolonged substance use. It helps determine how well an individual can interact with others and manage everyday life.

The AMT evaluates cognitive impairment through a series of 10 questions assessing orientation, memory, and attention, areas commonly affected by alcoholism. Chronic alcohol abuse is associated with deficits in memory, executive function, and attention, which the AMT can identify.

Together, mRS, MOHS, and AMT provide a comprehensive evaluation of both functional and cognitive outcomes in alcoholism, aiding in clinical decisions and rehabilitation strategies [20].

## Conclusions

This case underscores the challenges in managing Marchiafava-Bignami disease (MBD), particularly highlighting factors associated with poor prognosis. High clinical suspicion, early diagnosis, robust nutritional support, and comprehensive long-term follow-up are essential for effectively managing this complex disorder; however, outcomes remain guarded in some cases. Specific clinical and radiological signs may alert physicians to the potential for poor outcomes. A history of chronic alcohol use disorder in a young patient, a Glasgow Coma Scale score below 10 on presentation, and the presence of extracallosal lesions on imaging are indicative of a more critical clinical course as well as persistent neurological deficits with incomplete recovery. Such cases may warrant more aggressive management from presentation, higher levels of care, and earlier involvement of a multidisciplinary team for rehabilitation. Long-term follow-up may allow for close monitoring of recurrence of symptoms, in managing sequelae and ensuring ongoing nutritional support. This case stresses the need for a larger case series to further define the various prognostic factors as well as develop effective treatment approaches for MBD.
